# Does heavy mental workload affect moral sensitivity among critical care unit nursing professionals? a cross-sectional study

**DOI:** 10.1186/s12912-021-00662-8

**Published:** 2021-08-10

**Authors:** Hosein Zahednezhad, Nasrin shokrollahi, Reza Ghanei Gheshlagh, Pouya Farokhnezhad Afshar

**Affiliations:** 1 Department of Psychiatric Nursing and Management, School of Nursing & Midwifery, Shahid Beheshti University Of Medical sciences, Velenjak St, Shahid Chamran Highway, Tehran, Iran; 2 Masters of Science in Critical Care Nursing, Imam Khomeini Hospital Complex, Tehran University of Medical Sciences, Tohid Square, Tehran, Iran; 3 Spiritual Health Research Center, Research Institute for Health Development, Kurdistan University Of Medical sciences, Pasdaran Blvd., Sanandaj, Iran; 4 School of Behavioral Sciences and Mental Health, Iran University of Medical Sciences, Shahid Hemmat Highway, 1449614535 Tehran, Iran

**Keywords:** Moral Sensitivity, Mental Workload, nursing professionals, Critical Care Unit

## Abstract

**Background:**

Moral sensitivity creates the basic attitude in providing effective ethical care to patients. Heavy mental workload is a major concern of critical care nursing professionals, which could adversely affect nursing staff and patients. The present study aimed to investigate the effects of mental workload and some demographic variables on the moral sensitivity of critical care nursing professionals.

**Methods:**

This cross-sectional, descriptive-correlational study was performed on 181 nursing professionals working in the critical care units of Shahid Rajaei Cardiovascular Medical and Research Center in Tehran, Iran. Data were collected using a demographic questionnaire, the moral sensitivity questionnaire, and the NASA-task load index to assess mental workload. Data analysis was performed in SPSS version 22 using descriptive statistics, independent t-test, Pearson’s correlation-coefficient, and regression analysis.

**Results:**

The results of regression analysis yielded no statistical significant relationship between heavy mental workload and moral sensitivity of the critical care nursing professionals, while clinical experience had a positive, significant association with moral sensitivity.

**Conclusions:**

Although care nursing professionals experience a heavy mental workload in critical care units, it does not decrease their moral sensitivity. In addition, experienced nurses have higher moral sensitivity and lower mental workload. Therefore, it seems that nursing managers should pay special attention to the importance of employing experienced nurses along with younger nurses.

## Background

The provision of nursing care, especially in critical care units, is intertwined with situations that require ethical decision-making process[1]. An individual with moral sensitivity can identify the problems of patients in a timely manner and make the best decisions for patient care [2]. Critical care units have a higher nurse-to-patient ratio compared to other units; in critical care units, nursing professionals spend more time with patients and patient-care is entirely provided by the nursing professionals. The special conditions of the patients admitted to these units highlight the importance of moral decision-making in patient care [3]. Ethical sensitivity plays an important role in the ability to make moral decisions and is defined as the ability and capacity of individuals to make moral decisions [4].

Moral sensitivity is a combination of the awareness of moral dimensions (e.g., tolerance, calmness, responsibility, and consideration of ethical issues) [5]. Moral sensitivity is reflected in the concerns of an individual about his/her actions toward others, helping him/her to identify the correct course of action [6].

Research suggests that nursing professionals with higher moral sensitivity, in general, deliver higher quality of clinical care and have more respectful professional interactions with their colleagues [4].

Professional ethics is inherent to patient care; however, research suggests that the standards of professional ethics of conduct are susceptible to compromise, especially when the nursing staff are mentally and cognitively distressed[7].Research on the concept of moral sensitivity could be beneficial in developing appropriate strategies to promote moral sensitivity and subsequently increase the quality of nursing care and patient safety in healthcare organizations [7, 8]. In a study conducted among Chinese and Swiss nursing professionals, both groups were reported to experience moral issues due to the poor communication caused by work overload [9].

Nursing professionals are faced with heavier workload than ever before; the higher demand for health care, insufficient nurse staffing, and the subsequent increased overtime hours are among the contributing factors to the heavier workload of nurses [10]. It seems that the heavy workload of critical care nursing professionals is associated with inadequate nursing care ,patient dissatisfaction and the time that nursing staff allocate to various tasks [11].

In addition, heavy workload in critical care units could increase the mental workload ,fatigue, incidence of medical errors and decrease the quality of nursing care [12, 13].

Mental workload basically represents the capacity of the attention needed to fulfill occupational tasks. When these tasks exceed the capacity of the individual, they must adapt to the tasks or their performance would decrease [14, 15].Critical care nursing professionals are among the group of healthcare providers that are most likely to experience heavy mental workload [16]. Mental workload refers to the amount of mental efforts needed in performing a task [17, 18]. As the work environment of nursing profession is becoming increasingly complex, giving rise to higher mental workload [19]. Meanwhile, critical care nursing professionals are exposed to severe mental workload as they are constantly making important decisions regarding patients’ health and life [20].

Traditional theories of moral development emphasize the role of controlled cognition in adult moral judgment, while new theories emphasize the role of intuitive or automatic emotional processes. Utilitarian moral judgments are driven by controlled cognitive processes while non-utilitarian (characteristically deontological) judgments are driven by automatic emotional responses [21, 22].

There is a paucity of information about the impact of stressful work conditions on nursing professionals’ ethical decision making. Therefore, we implemented a cross-sectional study with the primary objective of assessing if based on utilitarian view, increased mental workload negatively affects moral sensitivity of critical care nursing staff. Our second objective was to evaluate correlation between demographic characteristics and moral sensitivity and mental workload of critical care nursing staff.

## Methods

### Research Design and Setting

In this cross-sectional, descriptive-correlational study, the correlations between moral sensitivity, mental workload, and a few demographic characteristics (age, gender, working hours per week, and clinical work experience) were evaluated in critical care nurses. The research setting was the adult intensive care unit of Shahid Rajaei Cardiovascular Medical and Research Center in Tehran, Iran. This academic cardiovascular center is one of the largest of its kind in the Middle East, where nursing professionals receive postgraduate training in cardiovascular nursing care.

### Participants

The nursing staff of the critical care unit of the hospital is 331 members strong, of whom, 181 were randomly invited to participate in this study. The sample size of 181 was calculated using Morgan’s table for sample size (N = 331, ß= 0.2, α = 0.05 and Probability = 0.5). The inclusion criteria of the study were: (1) a minimum of BSc degree in nursing; (2) a minimum critical care experience of one year; (3) informed consent for participation. The nurses randomly were selected via proportional sampling based on the inclusion criteria. To this end, a quota was allocated to each unit based on the number of the nurses in each unit and total sample size. Based on the allocated quota, the research samples in each unit were selected via simple random sampling.

### Measures

Data were collected using a demographic questionnaire, Lutzen’s standard moral sensitivity questionnaire (1994), and NASA-task load index (NASA-TLX) [23, 24]. The demographic questionnaire contained data on age, gender, marital status, clinical experience, and average working hours per week. The standard moral sensitivity questionnaire consisted of 25 items, which were scored based on a Likert scale, with the minimum and maximum score of zero and 100, respectively, and the higher scores indicated higher moral sensitivity. The translation and cultural adaptation of this scale in to Persian was performed by Borhani et al. (2013). The standardized reliability coefficient was measured by Cronbach’s alpha coefficient (α = 0.76)[25]. In the current research, the reliability of this questionnaire was determined by Cronbach’s alpha coefficient (α = 0.81).

The standardized NASA task load index (NASA-TLX) was used to measure the mental workload of critical care units nursing staff which provides an overall score of mental workload based on a weighted average of six subscales, namely mental demand, physical demand, time demand, performance, effort and frustration on a 20-point scale ranging from 0 (very low)to 20 (very high). The possible scores range from 0 to 100 [26].

The translation and cultural adaptation of this scale in to Persian was performed by Mohammadi et al. (2013). The standardized reliability coefficient was measured by Cronbach’s alpha coefficient (α = 0.84) [27]. In current study the reliability of the scale was confirmed at the Cronbach’s alpha coefficient of 0.81. The evaluation process was carried out in three stages of determining the weight of the load (6 items), determining the degree of the load (15 items), and determining the final score of the mental workload. In NASA-TLX, each subscale is divided into a range of 100 points with five-point steps, which are selected by the user in the first step, and their degree is determined by the user in the second step, so that an individual and personal weight are created by the pairwise comparison based on the importance of self-perception. In the final stage, the product of the weight score is multiplied by the scale score of each dimension and divided by 15 to yield the mental workload score (range: 0-100), higher scores indicating higher mental workload.

### Data Collection

After the approval of the research plan by the Research and Ethics Committee of Shahid Rajaei Cardiovascular Research Center, the lead researcher mailed the questionnaire packages to the 181 participants. A total of 11 nurses refused to participate in the study due to heavy workload; the researchers identified additional 11 nurses who met the eligibility criteria to take part in this study. The questionnaires were delivered to the selected nursing staff of each ward at the beginning of their work shift. Anonymous completed questionnaires were returned in sealed envelopes and were handed to the designated staff members at each department.

The mean time required to complete each questionnaire was estimated at 20 min. Sampling was performed during two months in 2018. After the completion of the questionnaires, the nurses placed the questionnaires in an envelope and handed the envelope to the secretary of their department.

### Statistical Analysis

Data analysis was performed in SPSS version 22.0 (SPSS; IBM, New York, NY) using Pearson’s correlation-coefficient (r) to assess the correlations between the study variables. In addition, Independent sample t-test was applied to compare the mean values of moral sensitivity and mental workload based on the gender and marital status of the participants. Multiple linear regression analysis (stepwise) was used to evaluate the predictive power of the independent variables for the dependent variables. All the statistical analyses were performed using two-tailed tests with an alpha error of 0.05, and the P-value of less than 0.05 was considered significant. Data were expressed as mean and standard deviation for the continuous variables and number (%) for the categorical variables. Prior to statistical analysis, the data set was screened for missing values. Preliminary data analysis revealed the existence of less than 2 % of missing values, median imputation was used for their replacement.

### Ethical Considerations

The study protocol was approved by the Research and Ethics Committee of Shahid Rajaei Cardiovascular Hospital (dated from 27/02/2017). Participation in the study was voluntary, and the respondents were informed of the research objectives, voluntary participation, anonymous responses, and confidentiality terms regarding their information.

## Results

The mean age of the subjects was 30.9 ± 4.9 years. Among the nurses, 138 (76.2 %) were female, and 94 (51.9 %) were married. The mean clinical work experience of the participants was 7.7 ± 4.7 years, and the mean working hours per week was 50.6 ± 8.5 h.

As shown in Table 1, the results of Independent sample t-test showed that gender and marital status were not significantly related to moral sensitivity of critical care nursing professionals (*P* > 0.05). Also, gender were not significantly related to mental work load of critical care nursing staff (*P* > 0.05), while mental workload of single nursing professionals were significantly higher than married staff (*P* < 0.05) (Table 1).

**Table 1:** Mean Scores of Moral Sensitivity and Mental Workload Based on Gender and Marital Status

Variables		N	Mean	SD	T	*P*-value	
Moral Sensitivity	Male	43	69.88	8.932	-0.934	0.173	
	Female	138	71.14	7.329			
Mental Workload	Male	43	63.298	10.6030	-0.620	0.999	
	Female	138	64.459	10.7582			
Moral Sensitivity	Single	87	70.75	7.260	-0.164		0.870
	Married	94	70.94	8.183			
Mental Workload	Single	87	67.287	9.4394	3.899		0.001
	Married	94	61.311	11.0451			

The obtained results indicated that moral sensitivity had a statistically positive significant correlation with clinical work experience (*r* = 0.20; *P* < 0.01), and had a statistically negative significant correlation with the mean working hours per week (*r* = 0.19; *P* < 0.01); as the mean working hours per week decreased and clinical work experience increased, the moral sensitivity of the nurses increased. Furthermore, the results of Pearson’s correlation showed that mental workload had a statistically negative relationship with moral sensitivity (*r*= -0.18; *P* < 0.05) and clinical work experience (*r*= -0.56; *P* < 0.01) and had a statistically positive correlation with mean working hours per week(*r* = 0.30; *P* < 0.01); as the clinical experience increased and mean working hours per week decreased, the mental workload decreased. In addition, as the mental workload of the nurses increased, their moral sensitivity decreased (Table 2).

**Table 2:** Means, standard deviations, and correlation matrix for study variables

Variables	M	SD	1	2	3	4
1-Moral Sensitivity	70.85	7.73	-			
2-Mental Workload	64.18	10.70	− 0.18*	-		
3-Weekly Working Hours	50.6	8.5	0.196**	0.30**	-	
4-Clinical Experience(Years)	7.7	4.7	0.20**	-0.567**	-0.48**	-

*P > 0.05 ** *P* > 0.01

At the next stage, Stepwise regression analysis was employed to determine the predictive power of the variables that were the significant predictors of moral sensitivity in the critical care nursing staff. According to Pearson correlation results, mental workload, clinical experience, and mean working hours per week were significantly related to moral sensitivity; therefore, the three variables were included in the regression model to predict moral sensitivity in critical care nursing staff. The results of the regression analysis indicated that between the variables of mental workload, clinical experience, and mean working hours per week, only the variable of clinical experience was included in the regression model and significantly predicted moral sensitivity (F = 7.622, *P* = 0.006). Clinical experience explained 20 % of the variance of moral sensitivity (Table 3).

**Table 3:** The results of Stepwise multiple regression

Model	Source of changes	SS	df	MS	F	R	R2	Adj R2	P
Clinical Experience	Regression	439.561	1	439.561	7.622	0.202	0.041	0.035	0.006
	Residual	10322.108	179	57.665					
	Sum	10761.669	180						

As shown in Table 3, regression coefficients indicate that one standard deviation change in clinical experience is associated with 0.20 standard deviation change in moral sensitivity of critical care nursing staff. Regression coefficients also show that clinical experience (p = 0.006) have a significant positive effect on moral sensitivity of critical care nursing staff. In other words, higher clinical experience is associated with higher moral sensitivity (Table 4).

**Table 4:** Stepwise regression analysis for variables predicting moral sensitivity in critical care unit nurse professionals

Model	Unstandardized Coefficients		Standardized Coefficients	t	P-value
	B	Std. Error	Beta		
Constant	68.288	1.085	-	62.95	0.001
Clinical Experience	0.332	0.120	0.202	2.76	0.006

## Discussion

The present study aimed to determine the correlations between mental workload, moral sensitivity, and some demographic variables in critical care nursing professionals and address the question of whether the high mental workload of critical care nursing professionals would decrease their level of moral sensitivity based on Utilitarian view. The results of regression analysis showed that despite the high mental workload of the nursing professionals, this concept was not significantly related to their moral sensitivity. The results indicated that nursing professionals in difficult working conditions and increasing mental workload also make decisions based on non-Utilitarian perspective and these conditions do not reduce their moral sensitivity.

In a study by De Casterlé et al. (2008), nursing staff viewed heavy mental workload as the most important influential factor in the reduction of moral sensitivity[28]. Mental workload is high in critical care units due to the need for quick information processing to make urgent decisions, many of which are moral decisions. Despite these limitations, the results of the present study indicated that the mental workload of the nurses was not significantly correlated with their moral sensitivity. The philosophy and nature of the nursing profession is such that moral sensitivity is always a priority and is even more important an issue than the therapeutic aspects of this healthcare profession [29]. Attention to patient benefit and faithfulness to ethics lead to positive psychological responses among nursing professionals, such as satisfaction, motivation, and competence [30].

According to the findings of the current research, the mean score of the moral sensitivity in critical care nursing professionals was higher than the average value. In a study conducted by Kim (2005) in Korea, clinical nursing staff had a moderate level of moral sensitivity to hospital issues, and the findings demonstrated no associations between marital status with moral sensitivity and its dimensions. In the study by Lutzen (2010) on moral sensitivity, no significant difference was reported between men and women in this regard [31], and Comrie (2005) reported no correlations between moral sensitivity and its dimensions with demographic variables [32]. In the current research, inverse correlations were denoted between moral sensitivity and the mean working hours per week. Accordingly, moral sensitivity decreased with the increased mean working hours per week, which was not confirmed in the regression equation analysis. In the study by Hariri (2011), the nurses who had more overtime hours (i.e., higher mean working hours per week) had lower scores of moral sensitivity [33]. High working hours per week may cause dissatisfaction in nurses, thereby diminishing their sensitivity to moral issues; in fact, high work pressure may cause indifference in nurses over time.

According to the results of the present study, higher clinical experience was associated with the increased moral sensitivity of the nursing professionals. Consistently, the findings of Lutzen et al. (2010) indicated that with increased work experience, the scores of moral sensitivity increased in nursing professionals. In addition, the study by Schluter (2008) showed that the individuals with more work experience (6–10 years) had higher moral sensitivity compared to those with less work experience (1–5 years). The study by Huang (2015) on Chinese nursing staff also demonstrated that low work experience was a barrier to moral sensitivity, and with increased experience, nurses became more sensitive to moral issues [34]. This could be attributed to the acquisition of professional and ethical skills, as well as the increased awareness of nursing professionals and their supportive and effective role in patient care, which increase their moral sensitivity with more work experience. An important contributing factor to solving moral issues by nursing professionals is reflection upon moral problems in the past, which could sustain its impact on solving the moral issues of nurses even up to 20 years [35]. In other words, the nursing professionals who have more work experience are more experienced in the management of clinical problems and moral issues. Therefore, they could handle such issues with more ease and have higher moral sensitivity compared to inexperienced nurses.

Critical care nursing professionals are more prone to heavy mental workload because they are constantly making important decisions regarding the health and life of patients [20]. The results of the present study indicated that the mean score of mental workload in the nursing professionals was 64.18 ± 10.70. In another research, Gaba (1990) and Nasirzad(2019) evaluated the workload of nursing professionals using NASA-TLX, reporting that the mean score of mental workload in critical care nursing professionals was 83.27 ± 11.3( and 70.21± (12.4) respectively [36, 37]. One of the reasons for the heavy mental workload of critical care nursing professionals may be the sensitivity of nursing profession duties in these units and the fact that any error may cause irreversible incidents for patients. In the study by Hoonakker et al. (2011) regarding mental workload in critical care nursing professionals, no significant difference was observed between different age groups in terms of mental workload. According to the study by Xiao (2011), mental workload decreased with age [38], and no significant correlations were observed between gender and mental workload and its dimensions. On the other hand, the studies by Malekpour (2013) and Hoonakker (2011) have shown that mental workload in female nursing professionals was significantly higher compared to male nurses [26, 39]. Furthermore, the findings of Xiao (2011) denoted that mental workload was higher in women compared to men. Despite the lack of a significant association between these variables in the present study, mental workload was observed to be higher in the female nurses compared to the male nurses. In addition, the total score of mental workload was higher in the single nursing staff compared to the married ones.

Our findings demonstrated a significant correlation between the total score of mental workload and mean working hours per week. In the study by Smith (2004), long working hours had a significant correlation with mental workload as increased working hours led to higher mental workload [40]. Heavy mental workload and long working hours play a key role in the incidence of fatigue. Although exhaustion may not necessarily lead to the discontinuation of work, the quality of work may decline as a result of this issue [41]. The fatigue caused by long working hours could also increase mental workload. According to the results of the present study, the mean working hours of the nursing professionals per week was high and could increase their mental workload.

The results of the present study showed a significant association between the total score of mental workload and clinical experience of the nursing professionals. Similarly, the study by Mohammadi (2011) indicated a significant correlation between clinical experience and mental workload. Mental workload could be influenced by prior experiences [14]. In other words, past experiences may reduce mental workload by changing skills or knowledge.

### Limitations of the Study

The data collection tool in this study was a self-report questionnaire, which might have caused social desirability bias. However, this issue might have had an insignificant impact on the findings since the questionnaires were completed by the participants anonymously, and the nurses delivered the questionnaires in an envelope during the data collection process. Current study was conducted only in critical care nursing professionals of a single hospital in Iran. Therefore, findings should be cautiously generalized to other settings, populations, and areas.

## Conclusions

Although the nursing professionals experienced heavy mental workload in critical care units, it did not decrease their moral sensitivity. It is a valuable finding that shows nursing professionals adhere to ethical principles even in the most difficult situations. Furthermore, the obtained results indicated that experienced nursing professionals had lower mental workload and higher moral sensitivity.

### Practical Implications

It seems that nursing managers should reduce the mental workload of young nursing professionals and promote their moral sensitivity by establishing proper mentoring programs and employing more experienced nursing professionals alongside these nurses. It is also essential that nursing managers plan standard working hours for nursing professionals considering the significant association between working hours and mental workload. So far, no research has been focused on the impact of mentoring programs on the moral sensitivity or mental workload of young nursing professionals, and this issue could be further investigated by nursing professionals researchers.

## Data Availability

The datasets used in the current research are available from the corresponding author on reasonable request.

## Abbreviations

NASA-TLX: NASA task load index
